# The Philani MOVIE study: a cluster-randomized controlled trial of a mobile video entertainment-education intervention to promote exclusive breastfeeding in South Africa

**DOI:** 10.1186/s12913-019-4000-x

**Published:** 2019-04-02

**Authors:** Maya Adam, Mark Tomlinson, Ingrid Le Roux, Amnesty E LeFevre, Shannon A McMahon, Jamie Johnston, Angela Kirton, Nokwanele Mbewu, Stacy-Leigh Strydom, Charles Prober, Till Bärnighausen

**Affiliations:** 1 The Department of Pediatrics, Stanford University, Stanford, CA USA; 2 Stanford University’s Digital Medical Education International Collaborative (Digital MEdIC), Cape Town, South Africa; 3 Stanford Center for Health Education, Stanford, CA USA; 4 Institute for Life Course Health Research, Department of Global Health, Stellenbosch University, Stellenbosch, South Africa; 5 Philani Maternal Child Health and Nutrition Trust, Cape Town, South Africa; 6 Johns Hopkins Bloomberg School of Public Health, Baltimore, USA; 7 Division of Epidemiology and Biostatistics, University of Cape Town School of Public Health, Cape Town, South Africa; 8 Heidelberg Institute of Global Health (HIGH), Medical Faculty and University Hospital, Heidelberg University, Heidelberg, Germany; 9 Stanford University, Stanford, CA USA; 10 Department of Global Health and Population, Harvard T.H Chan School of Public Health, Boston, USA; 11 Africa Health Research Institute (AHRI), Somkhele, KwaZulu-Natal, South Africa

**Keywords:** Mobile health, Video, Narrative, Entertainment-education, Community-based, Community health worker, Human-centered design, Maternal child health behavior, Breastfeeding, South Africa

## Abstract

**Background:**

In South Africa, rates of exclusive breastfeeding remain low and breastfeeding promotion is a national health priority. Mobile health and narrative entertainment-education are recognized strategies for health promotion. In-home counseling by community health workers (CHWs) is a proven breastfeeding promotion strategy. This protocol outlines a cluster-randomized controlled trial with a nested mixed-methods evaluation of the MObile Video Intervention for Exclusive breastfeeding (MOVIE) program. The evaluation will quantify the causal effect of the MOVIE program and generate a detailed understanding of the context in which the intervention took place and the mechanisms through which it enacted change. Findings from the study will inform the anticipated scale-up of mobile video health interventions in South Africa and the wider sub-Saharan region.

**Methods:**

We will conduct a stratified cluster-randomized controlled trial in urban communities of the Western Cape, to measure the effect of the MOVIE intervention on exclusive breastfeeding and other infant feeding practices. Eighty-four mentor-mothers (CHWs employed by the Philani Maternal Child Health and Nutrition Trust) will be randomized 1:1 into intervention and control arms, stratified by neighborhood type. Mentor-mothers in the control arm will provide standard of care (SoC) perinatal in-home counseling. Mentor-mothers in the intervention arm will provide SoC plus the MOVIE intervention. At least 1008 pregnant participants will be enrolled in the study and mother-child pairs will be followed until 5 months post-delivery. The primary outcomes of the study are exclusive breastfeeding at 1 and 5 months of age. Secondary outcomes are other infant feeding practices and maternal knowledge. In order to capture human-centered underpinnings of the intervention, we will conduct interviews with stakeholders engaged in the intervention design. To contextualize quantitative findings and understand the mechanisms through which the intervention enacted change, end-line focus groups with mentor-mothers will be conducted.

**Discussion:**

This trial will be among the first to explore a video-based, entertainment-education intervention delivered by CHWs and created using a community-based, human-centered design approach. As such, it could inform health policy, with regards to both the routine adoption of this intervention and, more broadly, the development of other entertainment-education interventions for health promotion in under-resourced settings.

**Trial Registration:**

The study and its outcomes were registered at clinicaltrials.gov (#NCT03688217) on September 27th, 2018.

## Background

Despite global progress in reducing maternal, newborn and child mortality, more than 5 million children, around the world, die before reaching their fifth birthday [1]. Rates of under-5 mortality are highest in sub-Saharan Africa and, in 2015, 45% of these deaths occurred in newborns. A majority of under-5 deaths are the result of preventable diseases, the incidence of which could be reduced by implementing health-promoting maternal-child behaviors, such as exclusive breastfeeding (EBF) [1–4].

The health benefits of EBF, especially in low- and middle-income countries (LMIC), have been well documented [5]. For the child, these include protection against infectious diseases, improved measures of intelligence and decreased risk of becoming overweight and developing diabetes. EBF also confers health benefits for the mother, including a reduced risk of breast and ovarian cancer [5]. Globally, EBF could save an estimated 823,000 under-5 lives each year, as well as preventing 20,000 maternal deaths from breast cancer. Yet, despite World Health Organization recommendations that children should be exclusively breastfed for the first 6 months of life, EBF rates hover at only 37% in LMIC [5].

South Africa has one of the lowest rates of EBF in the world [5]. Estimates of EBF rates under-6 months in South Africa range from 8% [5, 6] to the most generous estimate, 32% [7]. Furthermore, the percentage of children exclusively breastfed appears to decrease sharply over the first 6 months, from 44% of infants aged 0–1 month to 24% of infants aged 4–5 months [7]. The health problems that result are largely due to unsafe formula feeding and the widespread practice of introducing solid foods early (often before 3 months) into the infant’s diet [8]. In South Africa, some reports suggest that more than 70% of infants are given solid foods before reaching 6 months, the recommended age for initiating complementary feeding [8]. Unsafe formula feeding, including the use of bottles with poorly cleaned nipples, can lead to diarrheal disease [9] from pathogens entering the gut. Solid foods, introduced too early into a child’s diet, can also cause gastro-intestinal infections as well as nutrient deficiencies when these foods displace breastmilk without providing adequate nutrient density [10]. The limited availability of reliable national data on infant feeding in South Africa complicates the design and evaluation of infant feeding interventions [8]. However, systematic reviews of the literature suggest that large-scale interventions focused on educating mothers can increase the prevalence of EBF and decrease infant mortality [11–14].

The narrative entertainment-education (E-E) approach to health education appears to be a promising strategy for promoting health behavior change [15, 16]. E-E content endeavors to deliver health messaging through an entertainment media framework. A growing body of scientific evidence suggests that E-E is an effective approach towards positively influencing beliefs, attitudes and behaviors [15, 17–20]. Especially in populations with low motivation and/or a reduced ability for cognitively evaluating the intended health messaging, E-E may be a powerful health education strategy [15]. The characteristics of effective E-E include: a) appealing narratives, b) high production quality, c) persuasive messages that are unobtrusive and d) high potential for involvement or identification with the presenters or characters portrayed [15]. While E-E can take many forms, a video-based approach readily supports the harmonious integration of these characteristics.

Video-based content, optimized for mobile devices, may also facilitate broad dissemination of health education, especially as the global penetration of mobile technology increases. Recent advances in mobile messaging have already begun to facilitate the delivery of health messages at scale [21–23] and researchers are increasingly recognizing the potential for mobile phones and tablets to play an important role in health education interventions in LMIC [23–26]. South Africa is an example of a LMIC at the forefront of mobile health (mHealth) initiatives, due to its rapidly expanding penetration of mobile phones and national internet infrastructure [27–29]. National, maternal-child mHealth initiatives built around free, text-based SMS messaging have attracted global attention and been well received [30, 31]. General “tech-savviness” in South Africa continues to grow, with 37% of adults reporting smartphone ownership and 42% reporting daily internet usage in 2016 [32]. Active experimentation with WhatsApp, a widely used mobile communication tool that supports the transfer of videos, is also underway in many sectors, including health [33, 34].

Even for South Africans who do not yet have access to mobile phones, a feasible dissemination pathway lies in the delivery of educational videos by community health workers (CHWs) who bring teaching tablets to their in-home counseling sessions [35]. Interventions that support women in their homes have demonstrated efficacy in improving breastfeeding rates [36]. The Philani Maternal Child Health and Nutrition Trust [37] is an example of a successful community based organization, employing “mentor-mothers”, CHWs who offer in-home health promotion counseling to pregnant women and mothers within their neighborhoods. Data from both case studies and quantitative research suggest additional improvements in breastfeeding rates when synergistic interventions – for example, mass media interventions – are used to supplement successful community-based programs [36]. Developing interventions that boost the efficacy of community-based programs may be a critical step towards ending preventable child and maternal deaths by 2030 (as called for by the World Health Organization and UNICEF) [38]. Prior research in South Africa suggests that such synergistic interventions are even more effective when they are created in close collaboration with community-based programs, actively involving local stakeholders in the content creation process [39].

Applying a human-centered design (HCD) approach to the development of health education interventions may be a powerful way of tailoring these interventions to the specific settings and needs of their target communities, a priority identified in the 2016 *Lancet* Series on Breastfeeding [36]. The HCD approach has been described as “constructive, experiential and rooted in the needs and context of end-users of a product or service” [40], with the end goal of developing novel solutions to pressing problems. The creation of health education using an HCD approach involves several principles and practices that have been well characterized in the literature [41]. These include focusing on an empathic understanding of the target audience, creating content through a process of rapid prototyping, gathering of feedback and responsive iteration. Finally, the HDC approach is characterized by a relatively high tolerance for ambiguity and failure during the design process [40, 42].

Recent systematic reviews of mHealth interventions for improving maternal and neonatal health outcomes have called for: 1) strong experimental research designs including randomized controlled trials, 2) feasibility research, 3) government involvement and 4) integration of mHealth interventions into the healthcare system [23]. The Philani MOVIE intervention will be evaluated using a randomized-controlled trial, which follows a successful, qualitative feasibility study [35] involving the same population of CHWs who will be delivering the intervention in this trial. The 13-video Philani MOVIE intervention was created in collaboration with the Western Cape Department of Health, the South African National Department of Health, the Philani Maternal Child Health and Nutrition Trust and UNICEF, among other local stakeholders. By integrating the Philani MOVIE intervention into the successful, community-based Philani Mentor Mother Outreach Program [37], we aim to explore the potential for innovative, mobile video interventions to positively impact breastfeeding rates in South Africa, an important predictor of maternal and neonatal health outcomes.

### Aims

This study will achieve the following specific research aims: To

1. Establish the
  - Effectiveness of the Philani MOVIE intervention for increasing the practice of exclusive breastfeeding
  - Effectiveness of the Philani MOVIE intervention for improving other infant feeding practices and maternal knowledge about infant feeding
2. Determine the usefulness of human-centered design principles when applied to the development of mobile health interventions
3. Elucidate the mechanisms of intervention action and the acceptability of the intervention to CHWs.

## Methods

### Study setting and implementation partner

**Fig. 1:**
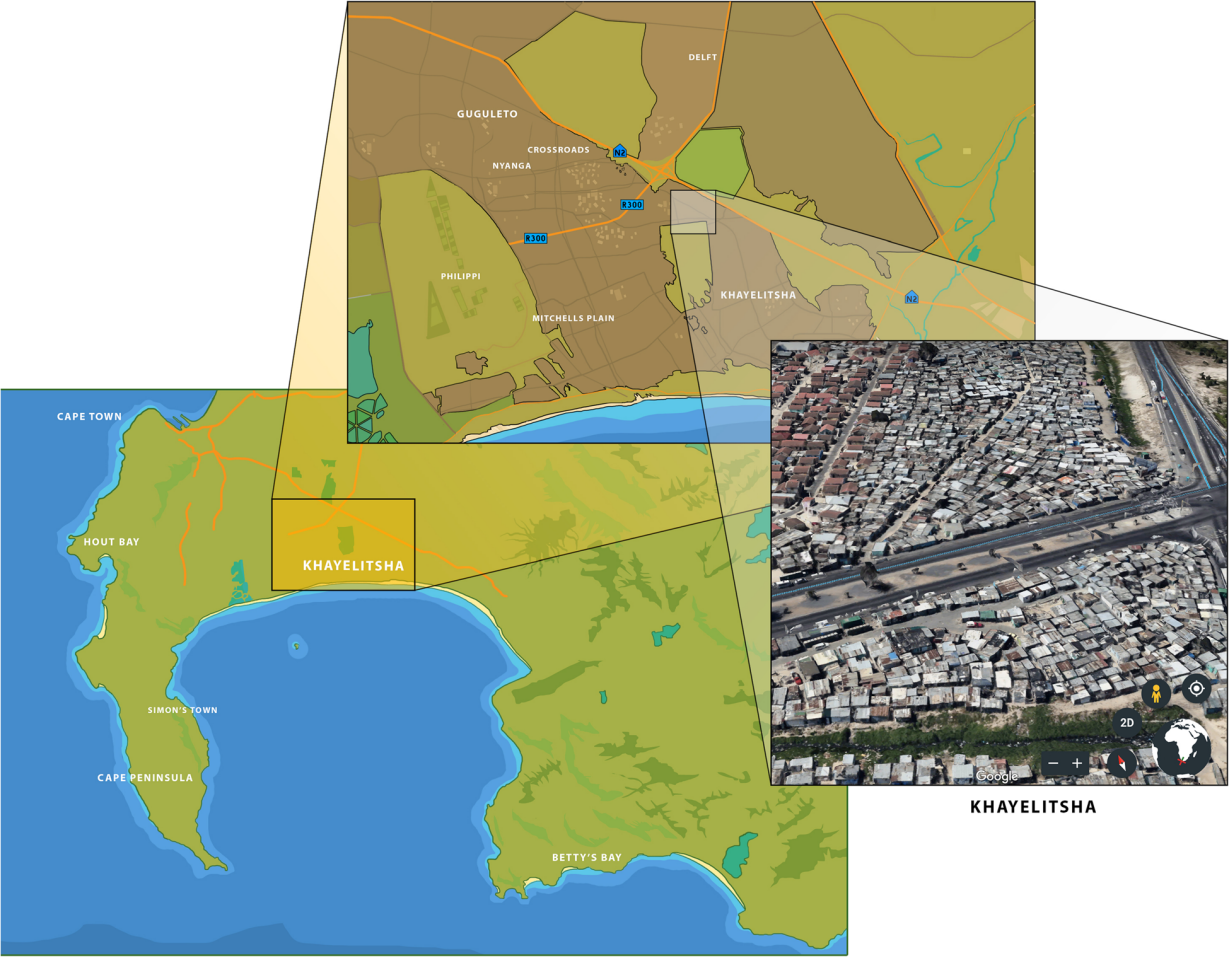
Regions of Operation of the Philani Maternal Child Health and Nutrition Trust, Western Cape, South Africa. (Illustrations by Shân Fischer. Map data© 2018 Google and© 2018 AfriGIS (Pty) Ltd.)

This study will be conducted within the under-resourced settlements of the Western Cape Province in South Africa. In 2010, the infant mortality rate in the Western Cape was reported to be 25 per 1000 live births [43]. A 2014 study of under-resourced communities in the Western Cape, found the EBF rate to be only 6% [44]. Operating within these communities (see Fig. 1), the Philani Maternal Child Health and Nutrition Trust [37] is a non-government organization serving approximately 100 under-resourced neighbourhoods. The Philani Mentor Mother Outreach Program is focused on improving child health outcomes through the deployment of “mentor-mother” CHWs. Mentor-mothers are identified as positive role models within their communities, after they have demonstrated success in raising healthy families despite resource shortages. Once recruited, mentor-mothers complete a standardized training program for delivering in-home health promotion counselling services within their communities. Among other health-promotion tasks, Philani mentor-mothers are trained to monitor growth, counsel parents around proper nutrition and refer sick family members to the nearest clinic when necessary. These mentor-mothers promote a variety of health behaviours, including those related to HIV-prevention, breastfeeding, child nutrition, growth and development [37, 45, 46].

### Description of the intervention

The Philani MOVIE intervention consists of 13 short, (2-5 min.) educational videos created over the course of 10 months in collaboration with community members and local stakeholders in maternal-child health. The health messaging in each video is closely aligned with the most recent WHO recommendations for infant feeding and narrated in English and isiXhosa, the languages spoken most widely among the study participants. The videos use simple syntax, avoiding medical jargon. Table 1 summarizes the video topics included in the Philani MOVIE intervention.

**Table 1:** Philani MOVIE video topics

1. Trailer 2. A Breastfeeding Story 3. The Benefits of Breastfeeding 4. How Breastfeeding Works 5. Breastfeeding Recommendations 6. A Kangaroo Mother Care Story 7. Common Challenges Faced by Breastfeeding Mothers 8. What Happened to the Practice of Breastfeeding? 9. Unsafe Infant Feeding Practices 10. Breastfeeding and HIV 11. Tips for Working Mothers who Breastfeed 12. When Breastfeeding Isn’t Possible 13. A Stunting Prevention Story	

### Intervention development

A human-centered design approach was applied during the development of the intervention. Formative feedback was solicited from end-users within the target audience as well as experts in multiple disciplines, including community medicine, academia, public health, media marketing and the performing arts. Scripts were collaboratively edited by content experts, who worked in parallel using Google Drive to allow for reliable version control. Early drafts of videos were then shared via WhatsApp with community members, most of whom did not use email regularly, informing a process of Rapid Iterative Testing and Evaluation [47, 48]. Feedback from community members was gathered via WhatsApp and during face-to-face “bodystorming” (physical brainstorming) sessions [47, 49] in which mentor-mothers tested the intervention through role-play activities involving mock-counseling sessions. Feedback was synthesized on a rolling basis, informing multiple iterations of the content. In this way, the health messages, language, illustrations, characters, color palate, narrative delivery style, logo and soundtrack were collaboratively and iteratively finalized over the 10-month content creation period. Key health and motivational messages were interwoven with animations created by local artists and underscored by the narratives of community mothers and South African celebrity mothers. Both community members and media marketing experts emphasized the importance of representing and celebrating South Africa’s diverse ethnic profile. This feedback shaped production decisions about both the illustrations included in the intervention and the profiles of the mothers featured in the intervention video footage. The trailer for the series can be previewed here https://youtu.be/1sss8ViPKJo [50].

### Trial design

Our study is a cluster-randomized controlled trial with baseline covariate adjustment and stratification [51] (See Fig. 1). The randomization unit is the mentor-mother. Each mentor-mother will enroll several pregnant women over the course of the trial. We conducted analyses of routine program data from these mentor-mothers and found that, among a range of available covariates, the only significant predictor of EBF was the type of neighborhood in which the mentor-mother lived and performed her health promotion duties. These neighborhood types include: informal, formal and mixed neighborhoods. We thus decided to stratify the randomization according to neighborhood type, to ensure balance of intervention versus control assignment by this covariate. Based on the existing literature [11, 52–59], we also expected that the following covariates would be associated with the primary outcome measure:

1. participant’s number of previous children
2. participant’s age
3. running water in the home
4. electricity in the home
5. participant’s employment status
6. participant’s education level

To further increase the statistical efficiency of our analysis, we thus decided to measure these covariates at baseline and adjust for them in our primary analyses, in addition to baseline neighborhood type. Randomization of the mentor-mothers was performed, using a computer-generated random allocation sequence, by faculty at Heidelberg University, Germany. The allocations were then transferred by email to Philani for implementation. Cluster randomization was preferred over individual randomization in this trial because each mentor-mother is responsible for counseling pregnant women within her neighborhood, making individual participant randomization logistically impossible without disrupting existing protocols.

#### Sample size calculation

**Fig. 2:**
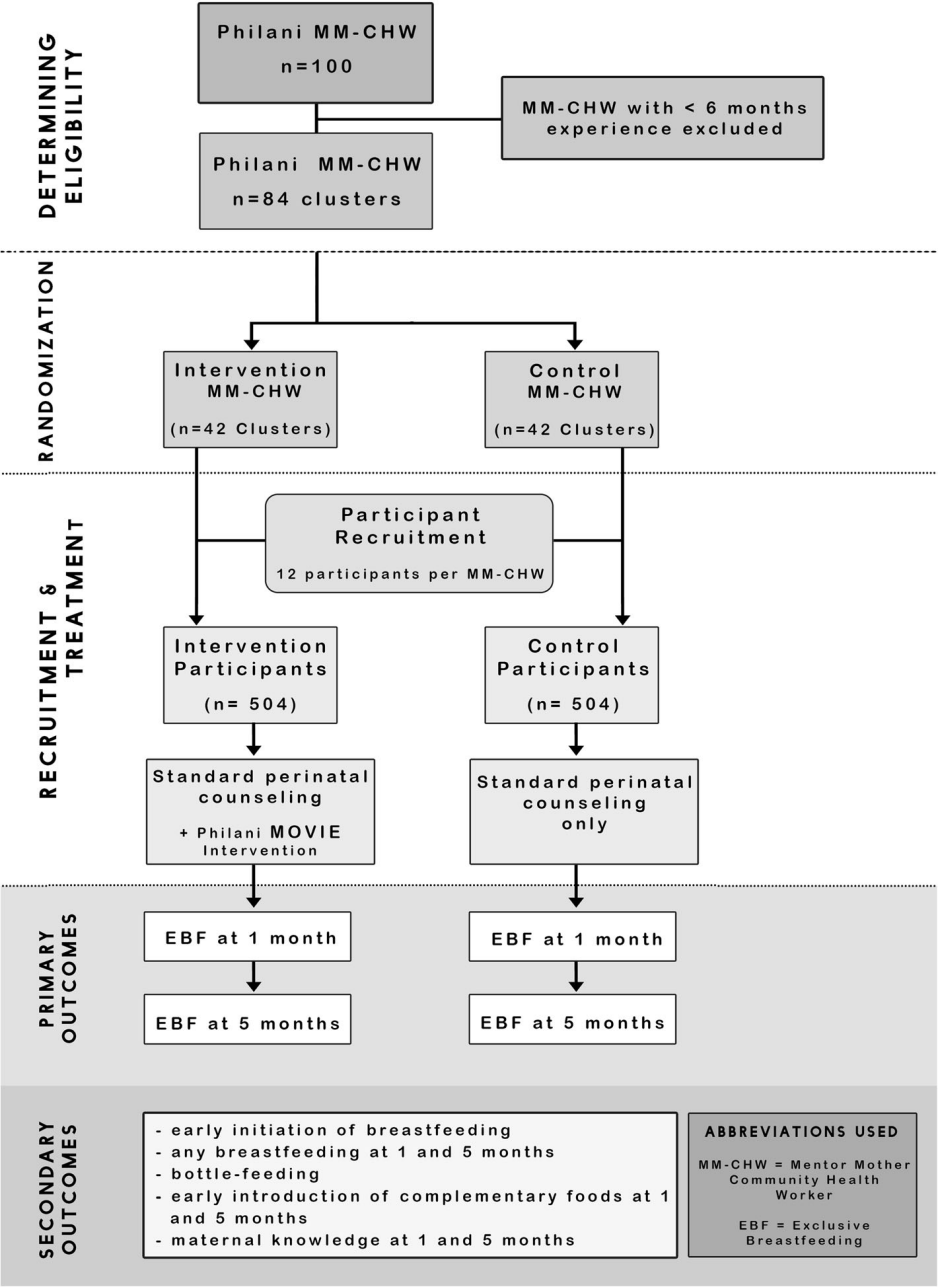
Trial Design for the Philani MObile Video Intervention for Exclusive breastfeeding (MOVIE) Study

Our sample size calculation was based on the primary outcomes: EBF at 1 and 5 months of age. We performed the calculations using standard methods for cluster-randomized controlled trials with baseline covariate adjustment and stratification [60]. The randomization units were the mentor-mothers. We assumed an intra-cluster correlation of our two primary outcomes of 0.1 [61]. We used routine program data describing the performance of the 84 mentor-mothers who will participate in this trial to inform our power calculation. Based on this data, and a range of other data sources on breastfeeding in South Africa [6–8, 36, 57, 62, 63], we assumed that 40% of mothers exclusively breastfed their infants at 1 month of age and 10% of mothers exclusively breastfed their infants at 5 months of age. Assuming a correlation between the baseline measurements and the primary outcome of 0.30 and an average enrolment of 12 pregnant women per mentor-mother over the course of this study, we estimated that at the 5% significance level the trial would have 80% power to detect a 13-percentage point increase in EBF at 1 months of age and a 9-percentage point increase in EBF at 5 months of age. This minimal detectable difference met our condition for policy relevance (an improvement of more than 15 percentage points). We thus set the total sample size for outcome assessment at 840 pregnant women plus 20% (to allow for loss to follow-up), i.e. 1008 pregnant women. Figure 2 illustrates the trial design.

#### Trial outcome measures

The outcome measures in this trial are based on the most recently published WHO indicators for the study of feeding practices in infants [64] and the most recent national infant feeding data for South Africa [7].

**The primary outcomes** will be short-term EBF (at 1 month) and long-term EBF (at 5 months). Primary outcomes will be measured using both point-in-time (24-h recall) and life-long (since birth) data, as recommended in the literature [65]. Primary outcomes data will be collected via tablet-based questionnaires and verified by follow-up telephone surveys, administered within one week of each tablet-based survey.

**Secondary outcomes** will include a) early initiation of breastfeeding (< 1 h after delivery, measured by recall on tablet-based survey at 1 month and verified by follow-up phone survey), b) *any* breastfeeding at 1 and 5 months (based on 24-h recall on tablet-based survey and verified by follow-up phone survey), c) bottle-feeding (based on 24-h recall on tablet-based survey and verified by follow-up phone survey), early introduction of complementary foods at 1 and 5 months (based on 24-h and since-birth recall, measured by phone surveys) and maternal knowledge at 1 and 5 months (measured by phone surveys).

Table 2 summarizes the quantitative outcomes that will be examined in this trial.

**Table 2:** Outcomes for the Philani MOVIE trial

Outcome Label	Outcome Definition	Method of Measurement
A. Primary outcome		
1. Short-term exclusive breastfeeding (24-h recall)	Infant, age 1 month, was exclusively breastfed in the past 24 h	These outcomes will be measured by tablet-based surveys, adapted from the South Africa Demographic and Health Survey 2016 [7] and following the most recent WHO indicators for infant feeding practices [64]. A point-in-time or current status measurement (based on 24-h recall) is presently the most common method for measuring exclusive breastfeeding [65]. Responses to initial tablet-based surveys administered at 1 and 5 months will be verified by follow-up phone surveys, conducted by an independent, vetted phone survey research company, within one week of the initial data collection timepoint. Life-long (since birth recall) data on exclusive breastfeeding, will also be collected and verified by phone survey. Reporting indicators based on both point-in-time and life-long data has been recommended as a stronger approach than either of these in isolation [65].
2. Long-term exclusive breastfeeding (24-h recall)	Infant, age 5 months, was exclusively breastfed in the past 24 h	
B. Secondary outcomes		
1. Short-term exclusive breastfeeding (since birth recall)	Infant, age 1 month, has been exclusively breastfed since birth	
2. Long-term exclusive breastfeeding (since birth recall)	Infant, age 5 months, has been exclusively breastfed since birth	
3. Early initiation of breastfeeding	Infant was breastfed within the first hour of life (based on recall at 1 month)	This outcome will be measured in the 1 month tablet-based survey and verified by phone survey within one week of the initial data collection timepoint.
4. *Any* breastfeeding at 1 month	Infant, age 1 month, received *any* breastmilk in the past 24 h, even if not exclusively breastfed	These outcomes will be measured by tablet-based surveys at 1 month and 5 months, adapted from the South Africa Demographic and Health Survey 2016 [7] and following the most recent WHO indicators for infant feeding practices [64]. Responses to tablet-based surveys will be verified by follow-up phone surveys, conducted by an independent, vetted phone survey research company, within one week of the initial data collection timepoint.
5. *Any* breastfeeding at 5 months	Infant, age 5 months, received *any* breastmilk in the past 24 h, even if not exclusively breastfed	
6. Bottle-feeding	Infant, under 6 months of age, was fed using a bottle with a nipple in the past 24 h.	
7. Early introduction of complementary foods at 1 month (24-h recall)	Infant, age 1 month, has received complementary foods in the past 24 h.	These out comes will be measured by phone surveys at 1 and 5 months. Participants will respond to an infant feeding questionnaire, incorporating both point-in-time and life-long data, adapted from previously published infant feeding measurement tools [82–85] and informed by the WHO indicators for infant feeding practices [64].
8. Early introduction of complementary foods at 5 months (24-h recall)	Infant, age 5 months, has received complementary foods in the past 24 h.	
9. Early introduction of complementary foods at 1 month (since birth recall)	Infant, age 1 month, has received complementary foods at some point since birth.	
10. Early introduction of complementary foods at 5 months (since birth recall)	Infant, age 5 months, has received complementary foods at some point since birth.	
11. Maternal knowledge at 1 month post-delivery	Maternal knowledge of breastfeeding current recommendations and basic health principles relevant to infant feeding measured at 1 month post-delivery.	These outcomes will be measured by phone survey questionnaires on maternal knowledge about breastfeeding (developed by study team, adapted from previously published breastfeeding knowledge assessment tools [59, 86, 87]).
12. Maternal knowledge at 5 months post-delivery	Maternal knowledge of breastfeeding current recommendations and basic health principles relevant to infant feeding measured at 5 months post-delivery.	

#### Qualitative component

Two qualitative components will be nested within this RCT in order to capture the human-centered design process of the intervention development, contextualize the results of the trial and understand the mechanisms through which the MOVIE enacted change. The qualitative methods used in this study will include in-depth interviews (IDIs) and focus group discussions (FGDs). Sampling for both qualitative study components will be purposive [66] and, to the extent possible given financial and logistical constraints, qualitative data collection will continue until saturation and redundancy are reached [67, 68]. All qualitative research assistants will work under the direct supervision of the research team and will be trained on qualitative methods. Data will be collected in a language of the respondents choosing and following informed consent. As soon as possible following the conclusion of a qualitative data collection activity, debriefings between qualitative research assistants and the study lead(s) will occur [69]. Debriefings will allow the study lead(s) to gain rapid insights into the content of data and strengthen not only the skills of data collectors but also the quality and trustworthiness of data in real-time [69]. All qualitative data will be tape-recorded, transcribed, translated into English as necessary, and quality checked for consistency and accuracy.

#### In-depth interviews on human-centered approaches in Mobile video design

IDIs will be conducted with stakeholders engaged in the design of the MOVIE intervention from concept generation to early prototyping to final refinement. Stakeholders will include those whose insights effectively shaped the content and delivery of the intervention including: Department of Health officials, representatives from governmental, non-governmental and UN agencies, health staff employed by Philani, film producers, graphic designers, marketing specialists, donors and academic faculty experts. Sampling for IDIs will be done via the snowball method wherein the study team will approach the Department of Health and key programmatic personnel engaged in the conception and development of the MOVIE intervention videos. These individuals will assist the research team in identifying initial respondents. These initial respondents will then be asked to assist in the identification of other respondents, who might facilitate an understanding of how and why the intervention was modified and refined in the course of video development.

#### Focus group discussions to contextualize and interpret quantitative findings

FGDs will be conducted with mentor-mothers following analysis of the quantitative data as a means to “explain” and thus to better interpret the quantitative results [70]. FGDs will serve as an opportunity to explore the heterogeneity in effects we expect to observe across mentor-mothers and the communities they serve, and to capture information about contextual elements that foster or hinder change. This information may prove particularly informative in the context of a later national rollout, wherein knowledge about the conditions and characteristics necessary to produce change will be actively sought. The type of purposive sampling to be employed will be criterion sampling wherein characteristics that emerge as relevant based on the quantitative results (such as mentor-mother age, place of residence or years of service) will shape the nature of the sample for the qualitative study [66].

#### Data collection and pre-trial training

Mentor-mothers in both the intervention and control groups will undergo training on how to obtain written, informed consent, record baseline variables and collect data on infant feeding practices directly on their tablets. This data will be collected as close as possible to the 1 month and 5 months post-delivery timepoints, (+/− 2 weeks) via infant feeding questionnaires. These questionnaires have been translated into isiXhosa and audio-recorded to help overcome literacy barriers. They will be filled out directly on the tablets by the participants. To standardize the collection of data, all 84 mentor-mothers will carry tablets with the infant feeding questionnaires for the duration of the study period. Only half [42] of the tablets will be loaded with the Philani MOVIE video intervention. All mentor-mothers will be trained on how to charge, use, troubleshoot and care for their tablets. These tablets are 8″ Android devices with 16GB of storage. The videos can be played offline and questionnaires completed and stored on the tablets while a mentor-mother is conducting home-visits in areas without internet access. Once the mentor-mother re-connects to the internet, all completed questionnaires will automatically be forwarded to the local research staff, based in Cape Town, for de-identification, cleaning and analysis.

Each tablet-based survey will be followed, within one week, by a phone-based survey conducted by Social Surveys, a professional telephone research company, located in South Africa. The use of telephone surveys for verification and follow-up after initial face-to-face interviews has demonstrated significant benefits. A dual sampling frame approach (using a combination of face-to-face and telephone interviews) has been recommended for such surveys, particularly for low-income and educationally under-resourced respondents [71–73]. Computer-assisted telephone interviewing (CATI) will also be employed to enhance accuracy of data collection and reporting. All data will be de-identified by local research staff in South Africa and carefully stored in the locked, secure offices of the local research team. Only de-identified data will be shared with collaborating research partner institutions for data analysis purposes.

Mentor-mothers in the intervention group will receive additional training on how to deliver the Philani MOVIE videos, which are designed in a modular fashion such that they can be viewed in any order, and thus tailored to the needs of the participant. Video sequencing decisions will be made by the mentor-mothers, who are trained by Philani to facilitate close alignment between health messages delivered during home-visits and the individual needs of each participant at each home visit and perinatal stage. Mentor-mothers in the intervention group will be instructed to administer each of the 13 intervention videos at least once to each participant during the study period. Video views will be tracked directly on the tablets and recorded on a paper-based tracking form by the mentor-mothers. The Philani MOVIE intervention will be delivered in parallel to the Standard Philani Perinatal Counseling Program (SoC), which includes counseling on infant feeding (see Table 3).

**Table 3:** Topics Addressed in Standard Philani Infant Feeding Counseling Sessions

1. Maternal beliefs about feeding 2. The practice of exclusive breastfeeding 3. The benefits of breastfeeding and the composition of breast milk 4. How milk is produced and released from the breast 5. How to position and attach a baby for breastfeeding 6. How to express breast milk 7. Common feeding difficulties and breast conditions relating to breastfeeding 8. Infant feeding for HIV positive mothers 9. Safe infant feeding for women who meet have chosen not to breastfeed and meet the AFASS criteria for formula feeding (Acceptable, Feasible, Affordable, Sustainable and Safe)	

#### Trial participants and recruitment

Participants in the trial will be 84 clusters of consenting, adult, pregnant clients (at least 1008 participants aged 18 years or more), living within the neighborhoods of 84 resident mentor-mothers and enrolled in the Philani Mentor Mother Outreach Program. Philani mentor-mothers are assigned to work only in the neighborhoods in which they live, resulting in 84 clusters of participants. Mentor-mothers with less than 6 months of experience and participants under 18 years of age will be excluded from the study. Based on Philani estimates, each mentor-mother typically identifies and enrolls 2–3 pregnant women per month within her neighborhood. Study participants will be enrolled on an ongoing basis until 1008 participants are enrolled. Written, informed consent will be collected from all study participants by a trained mentor-mother, prior to the collection of any data. Participants will be made aware that they have the right to withdraw from the study at any time. The study will conclude when the babies born to the enrolled participants during the study period have reached 5 months of age. Eligible pregnant participants will be recruited after their 20th week of pregnancy.

#### End-line focus group participants

End-line focus group participants will be the 42 mentor-mothers randomized to the intervention arm of the study. These mentor-mothers will be invited to take part in this explanatory, qualitative portion of the study after they have gained experience administering the Philani MOVIE intervention for 12 months.

#### In-depth interview participants

IDI participants will be consenting participants from the group of content experts, local stakeholders and community members who participated in the Philani MOVIE content creation process. Interviews will be conducted with experts in community medicine, academia, public health, media marketing and the performing arts, as well as the community mothers who helped to create the intervention and are representatives of its target audience.

### Data analysis

#### Quantitative analysis of the trial results

Our primary analysis will be based on intention-to-treat (ITT) at the unit of the individual newborn/individual mother, with standard errors adjusted for clustering at the level of the unit of randomization, i.e., the mentor-mothers. We will adjust for baseline neighborhood type in addition to the six additional baseline covariates described above (participant’s number of previous children, participant’s age, running water in the home, electricity in the home, participant’s employment status, and participant’s education level).

We will use mixed-effects generalized linear models for our primary analysis, using a fixed effect for the assignment to treatment vs. control arm and a random effect for mentor-mother. In particular, we will use modified Poisson models (generalized linear models with Poisson distribution and a logarithmic link function), for our binary outcomes, i.e., the primary outcome, as well as secondary outcomes 1–6 (see Table 1). We chose modified Poisson models because they generate estimates of risk ratios (and thus avoid the well-known interpretational difficulties associated with odds ratios) and because they converge more easily than alternative models that generate risk ratios (such as binomial models) [74]. For the continuous secondary outcomes (outcomes 11 and 12 – see Table 1), we will use generalized linear models with Gaussian distribution and identity link function.

#### Qualitative analysis

Debriefings following the conclusion of a qualitative data collection activity will corroborate and refine a template for thematic analysis [69]. In line with an underlying Grounded Theory approach, analysis will begin with a first reading of debriefing notes and transcripts to acquire familiarity with the data by the qualitative research team [75]. Categories and sub-categories will be developed, modified and expanded upon based on themes that emerge as analysis proceeds. An initial phase of open, inductive coding on a selection of rich, diverse and representative transcripts will be conducted. As new themes and theories emerge in the data, the research team will test theoretical notions and revisit earlier transcripts and debriefing notes in order to build a theory [75]. This analysis will be supported with software such as NVivo [76, 77]. In line with the principles of Grounded Theory, an extended literature review will follow the completion of coding [75].

### Theory of change

**Fig. 3:**
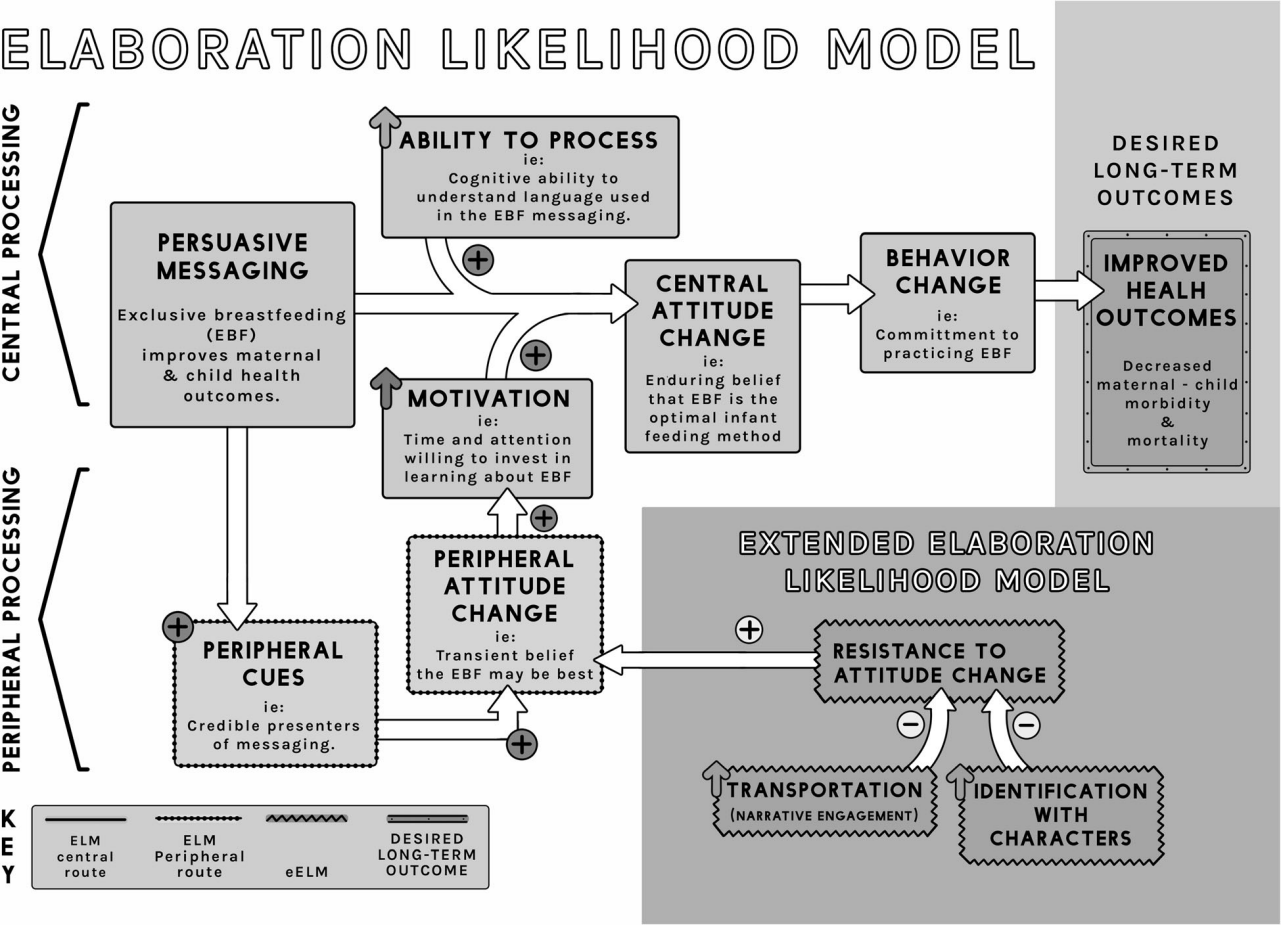
Intersection of the Elaboration Likelihood Model (ELM) and the extended ELM (eELM) theoretical models with desired long-term health outcomes

This work rests upon the theoretical underpinnings of the Elaboration Likelihood Model (ELM) [78], which suggests that there are two contributing pathways to achieving the changes in attitude that predict a desired behavioral outcome such as EBF. The first “central route” is influenced by the learner’s motivation and ability to cognitively process the information presented (ie: the health benefits of EBF). This can be influenced by factors such as the length of the content and the degree to which the language used is understood by the learner. The second “peripheral route” relies on cues embedded in the method of information delivery that contribute to its relative acceptability to the learner. Positive, peripheral cues, such as the learner’s subjective evaluation of the person delivering the information or the learner’s emotional involvement in the content, lead to peripheral attitude changes which, while less enduring than central attitude changes, can positively influence the learner’s motivation to process the messaging via the central route [78]. Both ELM and a variation on this model, called the extended Elaboration Likelihood Model (eELM) have been used to explain the efficacy of entertainment-education in changing leaner attitudes and behaviors in response to health messages and other types of persuasive messages [16, 19, 79]. According to the eELM model, effectively designed messages, containing positive peripheral cues, can reduce resistance to attitude changes (counter-arguing), when the learner is “transported” (engaged) in the narrative or identifies with its characters. This identification can be empathic or cognitive, through perceived similarity or wishful identification (a desire to emulate the characters featured). This model even suggests that the imagined “pseudo relationships” than can develop between viewers in the general public and on-screen celebrities, can be harnessed as a powerful peripheral cue to enhance attitude change in persuasive messaging [79]. Fig. 3 illustrates the intersecting theories of change on which our intervention is grounded.

### Study governance and oversight

This study will benefit from oversight by a data and safety monitoring board (DSMB), which will consist of a senior biostatistician, a senior local health systems researcher and a professor of pediatrics and health policy with expertise in primary care and health outcomes research. The DSMB will meet twice per year. It will evaluate the study progress and provide guidance on study continuation and any potential changes in study conduct in the case of unexpected events or operational difficulties.

## Discussion

The trial described in this paper aims to measure the causal effect of a mobile, video-based entertainment-education intervention, developed using human-centered design principles, on infant feeding behaviors in under-resourced South African communities. Nested qualitative components, involving interviews with local stakeholders and FGDs with mentor-mothers, will explore the development of the intervention and contextualize the results of the trial, while helping to characterize the mechanisms through which the Philani MOVIE intervention enact change.

The application of HCD principles is a new and promising complementary approach to the design of global health interventions [40]. A key principle of HCD is the idea that content should be created with the needs and context of the target community in mind. The intervention in this trial is based on a strong theory of change and was developed as part of a human-centered design study. Multiple cycles of formative feedback and rapid iteration generated an intervention that is intended to be rooted in the needs and context of the communities it addresses. Creating educational videos that are primarily visual, with carefully scripted audio tracks that convey health messages simply, can further align such content with the needs and context of its target community, especially in low-literacy settings. Additionally, translation of the content (into all 11 South African national languages) could further increase the scalability of the intervention and is underway. The organization of the intervention into short, modular videos was intended to optimize engagement and facilitate their flexible use and dissemination via multiple mobile technology pathways.

Applying the principles of community-based research [80, 81] to the design and production of this intervention facilitated the HCD approach to its development. We harnessed the strengths and resources within the target communities by soliciting their feedback on early prototypes and their active participation in the content creation process. The resulting intervention includes the stories and voices of local community mothers, presented alongside the parallel narratives of local celebrities. The decision to represent a variety of ethnicities, was made in an attempt to create an intervention that would resonate broadly across multiple South African demographics. The idea of including celebrities’ stories originated within the community and was implemented with the goal of emphasizing the common aspirations and challenges faced by many new mothers. These narrative elements were also included as positive peripheral cues, to optimize peripheral processing of health messages through realistic identification with community mothers and wishful identification with celebrity mothers. As a result, the intervention aims to inhibit resistance to attitude change (counter-arguing) as posited by the theoretical models underpinning this intervention. By partnering closely with target communities and local stakeholders during the intervention development, we were able to: a) identify and build on the strengths and resources within our target communities, b) collaboratively define priority problems and the desired characteristics of the intervention intended to address them and c) use an iterative-feedback process, with a high tolerance for failure and rapid reiteration, to create an intervention that is closely tailored to the needs and context of the target audience.

In the Philani MOVIE intervention, mobile and other emerging technologies facilitate the dissemination, but also the iterative HCD development of the content. The use of Google Drive to collaboratively edit scripts in parallel with version control and the ability to share drafts of video content with community members by WhatsApp facilitated a brisk production timeline for the intervention. The use of WhatsApp and other messaging tools to solicit rapid feedback at key decision points, even from community collaborators who were uncomfortable using email, may eventually prove to be a powerful addition to the production workflow for community-based health education content.

In addition to facilitating the content creation process, the growing penetration of smartphones, tablet technology and general tech-savviness in LMIC yield promising pathways for disseminating content to under-resourced communities. This dissemination can be a) mediated by community health workers or b) direct-to-learner approaches in future initiatives where every family has access to a smartphone or tablet. In anticipation of these emerging pathways, we find ourselves compelled to create a comprehensive, multi-lingual, free, accessible, engaging and impactful “video library for health”. Such a library would allow for timely updating, ongoing iterative improvement and broad dissemination of critical, preventive health education messages.

To this end, our research is intended to support capacity building for a “next generation” of digital, maternal-child health education, a generation of innovative educational tools, rooted in the needs and contexts of the audiences they intend to serve. By doing so, we believe we have an opportunity to support the adoption of behaviors that will form the cornerstone of healthier and more prosperous futures for mothers and children around the world. This cluster-randomized controlled trial serves to provide rigorous scientific evidence about the causal effectiveness of one particular intervention in this broader workstream. 

## Abbreviations

CATI: Computer-assisted telephone interview
CHW: Community health worker
DSMB: Data and safety monitoring board
EBF: Exclusive breastfeeding
E-E: Entertainment education
eELM: Extended elaboration likelihood model
ELM: Elaboration likelihood model
FGD: Focus group discussion
HCD: Human-centered design
IDI: In-depth interview
LMIC: Low- and middle-income countries
MM-CHW: Mentor-mother community health worker
MOVIE: Mobile video intervention for exclusive breastfeeding
RCT: Randomized controlled trial
SoC: Standard of care
UNICEF: United Nations International Children’s Emergency Fund
